# Propranolol, Promising Chemosensitizer and Candidate for the Combined Therapy through Disruption of Tumor Microenvironment Homeostasis by Decreasing the Level of Carbonic Anhydrase IX

**DOI:** 10.3390/ijms241311094

**Published:** 2023-07-04

**Authors:** Barbora Puzderova, Petra Belvoncikova, Katarina Grossmannova, Lucia Csaderova, Martina Labudova, Silvia Fecikova, Jaromir Pastorek, Monika Barathova

**Affiliations:** 1 Biomedical Research Center, Institute of Virology, Slovak Academy of Sciences, Dubravska Cesta 9, 845 05 Bratislava, Slovakia; barbora.puzderova@savba.sk (B.P.); katarina.laposova@savba.sk (K.G.); lucia.csaderova@savba.sk (L.C.); martina.labudova@savba.sk (M.L.); monika.barathova@savba.sk (M.B.); 2 National Institute of Lung Disaeses, Thorax Surgery and Tuberculosis, Vyšné Hágy 1, 059 84 Vysoké Tatry, Slovakia; silvia.fecikova@gmail.com; 3 Mabpro, a.s., Dubravska Cesta 2, 841 04 Bratislava, Slovakia; jaromir.pastorek@gmail.com

**Keywords:** carbonic anhydrase IX, propranolol, hypoxia, tumor microenvironment, hypoxia inducible factor 1, chemoresistance

## Abstract

Resistance to chemotherapy represents a persisting medical problem, ranking among main causes of chemotherapy failure and cancer mortality. There is a possibility to utilize and repurpose already existing therapeutics which were not primarily intended for oncological treatment. Overactivation of adrenergic receptors and signaling dysregulation promotes tumor progression, metastatic potential, immune system evasion, tumor angiogenesis and drug resistance. The non-selective beta-blocker propranolol, approved in infantile haemangioma treatment, has a high potential for use in cancer therapy. We analyzed the effects of propranolol and 5-fluorouracil combination on sensitive and resistant cells derived from colorectal carcinoma in monolayers, single-component and co-culture spheroids and in vivo mouse models. Our results revealed that propranolol is able to exert its effect not only in chemosensitive colorectal cells, but also in 5-fluorouracil resistant cells. Propranolol disrupts the hypoxic adaptation machinery by inhibiting HIF1α, carbonic anhydrase IX, and activates apoptosis, which may be important in the management of chemo-resistant patients. We showed that propranolol slows down the growth of xenografts formed from colorectal cancer cells, even from cells already adapted to the β-blocker. We provide clear evidence that blockade of β-adrenergic receptors affects essential signaling pathways modulating tumor microenvironment and thus the response to anticancer therapy. Our findings indicate that propranolol could be repurposed to serve as chemosensitizer in combined therapy aimed at disrupting homeostasis of tumor microenvironment.

## 1. Introduction

The role of catecholamines and beta-adrenergic signaling in tumor progression has been studied with increasing intensity. People often experience considerable emotional and psychological stress during cancer diagnosis and treatment [1,2]. Several studies show that chronic stress, anxiety and loss of social support in cancer patients lead to increased production of catecholamines associated with a poor response to therapy, formation of metastases, and cancer progression, including immunity impairment, modulation of inflammation, angiogenesis and invasion [3,4,5,6]. Catecholamines, adrenaline and noradrenaline, can subsequently pass from the surrounding normal tissue to the tumor. Beta-adrenergic receptors (ARs) are overexpressed not only in normal tissue but also in multiple cancer types, such as lung, colon, breast cancer. Signaling mediated by ARs contributes to tumorigenesis, tumor progression and metastasis [7,8].

Propranolol (C_16_H_21_NO_2_) is a non-selective antagonist of β1 and β2 adrenergic receptors. Initially, it was mainly indicated for the treatment of heart disorders and hypertension, but several other clinical therapeutic properties of this beta-blocker were gradually discovered. It is able to pass through the blood-brain barrier; it activates vasoconstriction and inhibits angiogenesis. Moreover, accumulated publications and clinical studies point to its possible role as a regulator of the tumor microenvironment, it can induce apoptosis, inhibit proliferation, angiogenesis, and metastasis across solid tumors [9,10,11]. Currently, propranolol is used as first-line therapy for infantile hemangioma [12]. In recent years, several studies have confirmed the antitumor effects of propranolol in colorectal cancer, breast cancer, glioblastoma, ovarian carcinoma, and pancreatic cancers [13,14,15,16].

Colorectal cancer (CRC) is the third leading cause of cancer related deaths in both genders worldwide, presenting a severe public health problem. CRC accounted for 10% of global cancer incidence in 2020 [17]. An important chemotherapeutic agent used for both adjuvant therapy and metastatic mCRC is 5-fluorouracil (5-FU), a fluorinated pyrimidine analog that requires intracellular conversion into active metabolites [18]. The cytotoxic mechanism of 5-FU involves the inhibition of thymidylate biosynthesis or misincorporation of fluorinated nucleotides into newly synthesized DNA or RNA [19]. The limiting factor in the clinical use of 5-FU is the innate or acquired chemoresistance present in almost 90% of patients with mCRC [20]. The combination of chemotherapeutics with different mechanisms of action could reduce the development of resistance [21]. Combined chemotherapy such as FOLFOX (folinic acid, 5-FU, oxaliplatin) and FOLFIRI (folinic acid, 5-FU, irinotecan) greatly improved the anticancer effect of 5-FU [22], but overall response rates still remain below 50% [23] due to the selection of chemotherapy-resistant cells. Several mechanisms play an important role in the development of chemoresistance. They include changes in the enzymatic activity of thymidylate synthase (TS), the fundamental enzyme of 5-FU metabolism, activation of the expression of drug transporters involved in multidrug resistance, induction of epithelial-mesenchymal transition (EMT), 5-FU-mediated epigenetic changes and microRNA (miR) dysregulations [24].

The tumor microenvironment (TME) has been recognized as an important modulator of the response to chemotherapy. Properties and composition of TME are linked to cancer progression, including metastasis, invasion, stemness, and acquired chemoresistance [25]. TME is a highly dynamic and heterogeneous network that includes both cellular and acellular factors regulating the homeostasis and function of normal and tumor cells. Physical and chemical characteristics of the microenvironment (low pH, hypoxia, high interstitial pressure, fibrosis) were identified as critical players [26,27]. Hypoxia represents a protective shield of tumor cells against chemo- and radiotherapy, promotes genetic instability, aggressive malignant phenotype, invasion, metastasis, reduces response to cytotoxins, supports resistance to apoptosis. Moreover, hypoxic TME is generally considered to be immunosuppressive, reducing the efficacy of immunotherapy [28]. Carbonic anhydrase IX (CAIX) is among the important proteins involved in TME regulation. It is a highly active transmembrane metalloenzyme [29,30], whose expression is activated in response to decreased oxygen concentration through the HIF-1 pathway. CAIX presence is limited to only few normal tissues, mainly to the gastrointestinal tract epithelia, however, CAIX is strongly associated with a broad range of tumors that either experience hypoxia or contain inactive pVHL [31]. CAIX reversibly catalyzes CO_2_ into bicarbonate by hydration. As a part of a transport metabolon, CAIX contributes to the maintenance of physiological intracellular pH and to the acidification of the extracelullar space, supports cell migration and invasion [32,33]. Activity of CAIX is dependent on the phosphorylation of threonine 443 in the intracellular tail by PKA [34]. CAIX is one of the best markers of tumor hypoxia, its expression in hypoxic tumors is associated with the aggressive tumor phenotype, metastatic tendency and poor response to conventional therapy. CAIX thus represents a suitable biomarker as well as a potential therapeutic target.

Characterization of new therapeutic combinations with 5-FU could represent an approach to avoid resistance. In this article, we follow up on our original results describing the effect of the non-selective beta blocker propranolol on the CAIX protein and consequently tumor cells [35]. Our goal was to monitor the effects of combined treatment with propranolol and 5-FU on sensitive and resistant cells derived from CRC in monolayers and 3D models of single-component spheroids, co-culture spheroids as well as in vivo mouse models.

## 2. Results

### 2.1. Preparation of 5-FU-Resistant and Propranolol-Adapted HCT116 Cells

**Figure 1:**
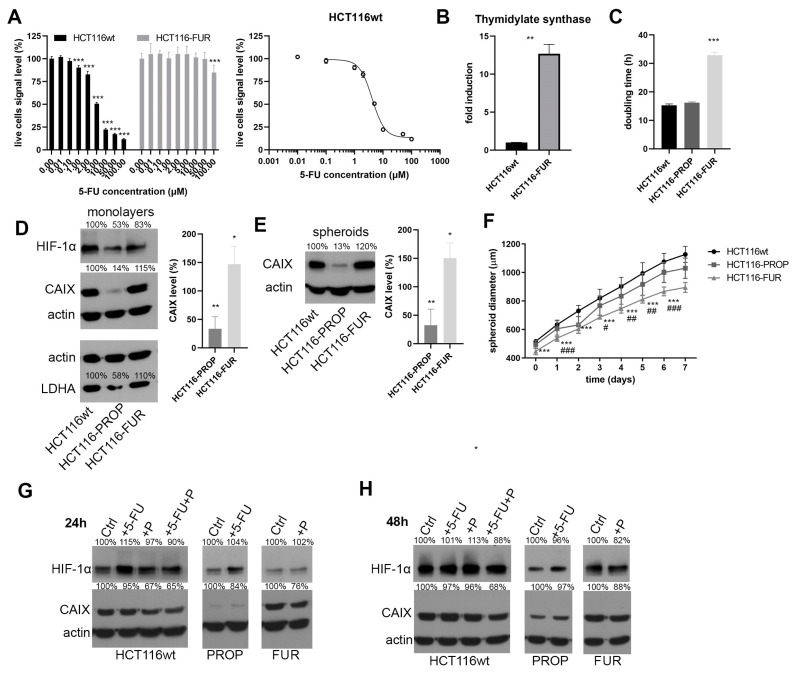
Characterization of 5-FU resistant and Prop adapted HCT116 cell lines and their response to treatments. (**A**) Viability of sensitive HCT116wt and resistant HCT116-FUR cells to increasing concentrations of 5-FU measured by Cell Titer Blue. Determination of IC50 concentration. (**B**) Thymidylate synthase mRNA, analyzed by RT-PCR, was increased in HCT116 5-FU cells. (**C**) Comparison of proliferation rates of HCT116 parental and derived cell lines measured by xCELLigence device. (**D**) Levels of HIF1α and its targets CAIX and LDHA in cell monolayers cultured in hypoxia for 48h detected by Western blot analysis. Densitometric analysis of CAIX levels in Prop adapted and 5-FU resistant cells expressed as percentage of the control HCT116wt (set as 100%) is given in the graph (n = 4, * denotes *p <* 0.05, ** *p <* 0.01). Prop adapted cell line displays reduced amounts of hypoxia adaptation machinery proteins. (**E**) Decreased CAIX level was confirmed in spheroids formed by HCT116-PROP cells. Densitometric analysis of CAIX levels from four independent experiments is shown in the graph (* denotes *p <* 0.05, ** *p <* 0.01). (**F**) Growth curves of spheroids formed by cells of parental and derived cell lines. (* and # denote significance of difference between wt and PROP spheroids, and wt and FUR spheroids, respectively). All graphs give mean ± stdev, * or # denote *p <* 0.05, ** or ## *p <* 0.01, *** or ### *p <* 0.001 (**G**,**H**) Effect of treatments of Prop (P), 5-FU, and their combination on cell monolayers cultured in hypoxia for 24 and 48 h. Protein levels were determined by Western blot analysis. The bands were densitometricly quantified in ImageJ software v1.41g and results were normalized to actin. Changes are expressed as percentage (denoted in the image) in comparison to the control set as 100%.

We prepared a cell population resistant to 5-FU (HCT116-FUR) from the colorectal cancer cell line HCT116 by gradually increasing the 5-FU concentration from 1 μM up to 30 μM. Results of Cell Titer Blue assays of the sensitive and resistant line confirmed that HCT116-FUR cells survive at the concentration of 30 μM, which is 7-times the IC50 value determined as 4.165 μM (Figure 1A), while the viability of sensitive parental cell line drops to 20% at 10 μM. Cell resistance to 5-FU can manifest by several mechanisms. One of them is the activation of thymidylate synthase (TS) expression. TS is the key enzyme for de novo synthesis of 2′-deoxythymidine-5′-monophosphate (dTMP) in the early stages of DNA biosynthesis. 5-FU acts as an inhibitor or antimetabolite that irreversibly inhibits TS by competitive binding [36,37]. The qPCR results confirmed that HCT116-FUR cells express a significantly higher TS level than sensitive HCT116wt (Figure 1B), which is characteristic of resistant cells but also of tumors, where TS status is one of the factors determining the success of 5-FU chemotherapy [38,39].

We also prepared a population of cells adapted to the beta-blocker propranolol (Prop) by continuous culture of HCT116wt cells in the presence of 50 μM Prop (HCT116-PROP). We thus simulated the situation in patients who chronically use propranolol as a result of comorbidity treatment. The cells did not show any changes in morphology during culture.

### 2.2. Characterization of HCT116-FUR and HCT116-PROP Cells Cultured in Monolayer and 3D Spheroid Model

Proliferation rate measurements using xCELLigence device showed a slower growth rate of HCT116-FUR (Figure 1C) in hypoxia. Using immunodetection, we analyzed possible changes in the expression of the CAIX protein, which represents an important part of the TME and of cellular adaptative machinery to hypoxia (Figure 1D). We detected a markedly lower CAIX protein level in cells adapted to Prop (Figure 1D) compared with parental HCT116 cultured in hypoxia (1% O_2_) for 48 h. The level of the regulatory subunit HIF1α also decreased. On the contrary, we observed an increase in CAIX level in cells resistant to 5-FU. The same trends of changes were observed for another target of the HIF1 transcription factor, lactate dehydrogenase A (LDHA) (Figure 1D).

As in vitro 3D cell models better mimic the spatial architecture, direct, and indirect cell communication and chemical tumor microenvironment—gradients of pH, oxygen, nutrients and waste products, we analyzed CAIX level in spheroids formed from HCT116wt, and prepared HCT116-FUR and HCT116-PROP cells. Our results confirmed (Figure 1E) a significant decrease in CAIX protein level in HCT116-PROP and its increase in HCT116-FUR cells. We also investigated whether adaptation to Prop or resistance to 5-FU affects the growth of spheroids. Growth curves (Figure 1F) demonstrate that spheroids formed by HCT116-FUR have a significantly smaller diameter and grow more slowly than spheroids formed from parental HCT116 cells. Size of HCT116-PROP spheroids is similar to HCT116wt spheroids, with their growth being only slightly slower.

### 2.3. Effect of Acute Action of Propranolol and 5-FU on HCT116-FUR and HCT116-PROP Cells

We monitored the effects of the acute action of 5-FU and Prop on the level of HIF1α and CAIX in cell monolayers cultured in hypoxia for 24 h and 48 h. The exposure to Prop (50 μM) indicated a decrease in CAIX level (Figure 1G) in parental as well as resistant cell lines. The greatest effect was observed with the combined action of Prop and 5-FU, where the decrease in the CAIX level was the most pronounced. Similar changes were detected at 48 h (Figure 1H). Our subsequent analyses were focused on more physiological spheroid models with 3D architecture where long-term effects of treatments can be monitored.

### 2.4. Influence of Acute Treatments of Prop and 5-FU in Spheroids

**Figure 2:**
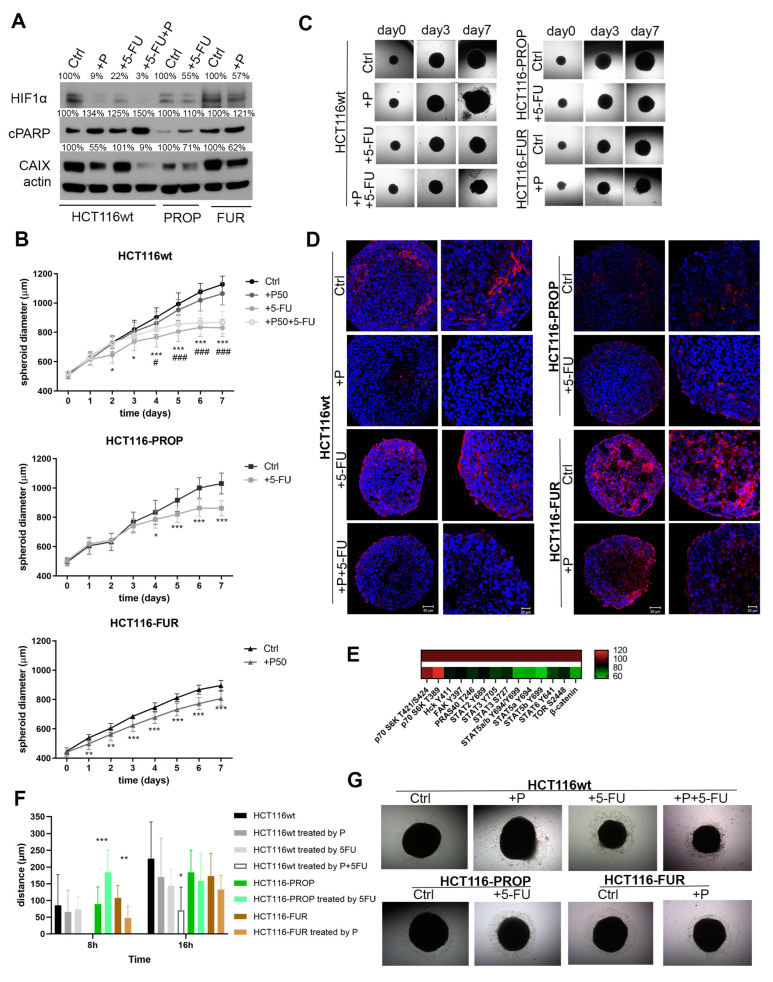
Response of single component spheroids formed by parental, Prop (P) adapted, and 5-FU resistant cells to acute treatments. (**A**) Western blot analysis of HIF1α and CAIX protein levels in control spheroids and spheroids treated by Prop and 5-FU. Apoptosis was determined by increase in cleaved PARP (cPARP) level in treated spheroids. The bands were densitometricly quantified in ImageJ software and results were normalized to actin. Changes are expressed as percentage (denoted in the image) in comparison to the control set as 100%. (**B**) Growth curves of control and treated single component spheroids. Graphs give mean ± stdev (n = 16). * and # denote significance of difference between Ctrl and 5-FU treatment, and Ctrl and combined treatment in HCT116wt spheroids. In all graphs: *, # denote *p* < 0.05, ** *p* < 0.01, ***, ### *p* < 0.001. (**C**) Representative images of the same spheroids after different periods of treatment acquired by transmitted light microscopy. (**D**) Images of immunofluorescent staining of CAIX protein in spheroid sections of single component spheroids acquired by confocal microscopy. (**E**) Analysis of the phosphorylation levels of selected kinases in HCT116wt spheroids treated/untreated by Prop detected by Proteome Profiler Array. Changes after Prop treatment (bottom row) are given as % of the untreated control set as 100% (upper row). (**F**) Evaluation of migration of cells from spheroids after their adhesion to the substrate. Before adhesion spheroids were treated with 5-FU, Prop, their combination or left untreated (Ctrl) for the period of 5 days (**G**) Representative images of cells which migrated from the treated and untreated spheroids after 48 h.

Spheroids were cultured in the presence of 5-FU and Prop separately or in combination for 7 days after their 4-day formation in hanging drops. Western blot analysis confirmed that Prop caused a significant decrease in CAIX and HIF1α levels also in spheroids (Figure 2A), including those formed from 5-FU resistant cells which can be of fundamental importance in the treatment of 5-FU resistant tumors. All the treatments led to increased apoptosis detected by apoptotic marker cleaved PARP in all cell lines, the most pronounced effects were observed after the combined action of 5-FU and Prop.

Growth curves demonstrate the effect of Prop and 5-FU on cell proliferation in spheroids. After treatment with Prop, 5-FU, and especially their combination, the growth of spheroids was slowed down (Figure 2B). Notable differences began to appear after 72–96 h of treatment. Treatments led to changes in the morphology of spheroids and to partial damage to spheroid integrity, especially in case of Prop and combined treatment of HCT116wt spheroids (Figure 2C). HCT116-FUR spheroids were not only the smallest, but also the most compact. We observed alterations in their growth already after 24 h of treatment with Prop. Blocking β-adrenergic pathways resulted in the removal of dead cells from spheroids.

### 2.5. 5-FU Changes CAIX Localization in Spheroids

Using immunofluorescence, we analyzed CAIX localization in spheroid sections treated with Prop, 5-FU, and their combination for 7 days. While the typical hypoxic/peri-necrotic expression of CAIX protein was preserved in the control spheroids formed from the parental HCT116wt line, the CAIX level was markedly decreased by the action of Prop in accordance with WB results (Figure 2D). Under the influence of 5-FU, we observed a change in the CAIX localization and its shift to the surface areas. A similar pattern of CAIX surface positivity was observed in spheroids treated by the combination of Prop and 5-FU. However, the total level of CAIX was very low, consistently with the CAIX immunodetection results. Sections of spheroids formed from HCT116-PROP displayed the stan-dard localization of CAIX within hypoxic regions comparable to that of CAIX in control HCT116wt spheroids. Due to the action of 5-FU, the signal was again transferred to the surface layers. We observed the most pronounced changes in CAIX localization in sections of spheroids formed from 5-FU resistant cells. We detected membrane CAIX diffusely throughout the spheroid volume, the CAIX level was high and corresponded to immunodetection results. After Prop treatment, the CAIX level decreased significantly. Using proteome profiler analysis of single component spheroids of HCT116wt we detected reduced phosphorylation of STAT family members, FAK, PRAS40, Hck and TOR, and decreased beta-catenin level in spheroids treated with Prop (Figure 2E). On the other hand, phosphorylation of p70 S6K increased.

### 2.6. 5-FU and Prop Treatments Suppress Cell Migration from Spheroids

Using Cell Observer, we investigated a possible impact of 5-FU and/or Prop treatment on cell migration from spheroids (Figure 2F,G). Treatment of parental HCT116wt cells with Prop and 5-FU independently slowed down the migration of cells from spheroid. The combined treatment delayed the process of spheroids adhesion to the plate surface and subsequently we observed a significantly reduced migration ability of cells. In the first 8 h of 5-FU exposure in spheroids adapted to Prop, we observed an increased migration, but the migration gradually slowed down. We also confirmed the reduced ability of cells to migrate from HCT116-FUR spheroids treated with Prop. When comparing the migration abilities of cells from HCT116-FUR, HCT116-PROP and HCT116wt spheroids, we found that 5-FU-resistant cells displayed the highest migration rate. The morphology of migrating cells is shown in Figure 2G.

### 2.7. Effect of Prop and 5-FU on Spheroids Co-Cultured with Fibroblasts

**Figure 3:**
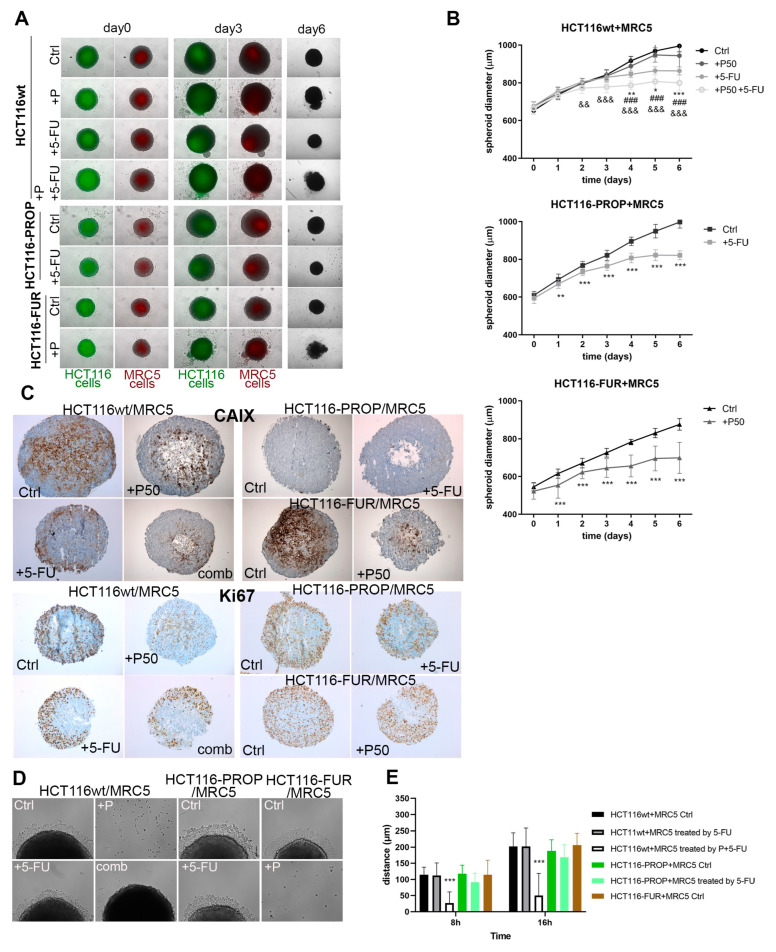
Effect of 5-FU and Prop treatments on co-culture spheroids formed from HCT116 parental and derived cell lines and MRC5 fibroblasts. (**A**) Images of control and treated co-cultured spheroids composition and morphology at different timepoints. Before co-culture HCT116 and MRC5 cells were labeled with Cell Brite Orange and Green dyes, respectively. Images at day 0 (beginning of treatment) and day 3 are composites of transmitted light and fluorescence microscopy at 10× objective. Images at day 6 were taken by 5× objective at transmitted light. Treatment led to growth retardation and spheroid damage accompanied by release of dead cells. (**B**) Growth curves of control and untreated co-culture spheroids displaying means ± stdev (n = 24). *, #, & denote significance of difference between Ctrl and Prop treatment, Ctrl and 5-FU, Ctrl and combined treatment in HCT116wt spheroids. (**C**) Representative images of immunohistochemical staining of CAIX protein and proliferation marker Ki67 in spheroid sections. (**D**,**E**) Analysis of the effect of treatment on the migration of cells from co-culture spheroids adhered to the surface. In all graphs: *, denote *p <* 0.05, **, && *p <* 0.01, ***, ###, &&& *p <* 0.001.

TME plays an important role in the process of acquiring resistance to chemotherapy. In single-component spheroids, we analyzed the effect of oxygen gradient, pH, nutrients in combination with 5-FU and Prop. Non-tumor components of the TME, including tumor-associated fibroblasts, influence the behavior of tumor cells and their response to therapeutic modalities through paracrine signaling, therefore we prepared co-culture models of 2-component spheroids formed by colorectal tumor cells and MRC5 fibroblasts combined in the ratio of 1:1. Before co-culture in ULA plates, epithelial cells and MRC5 were labeled with CellBrite Green and CellBrite Orange fluorescent labels, respectively. The presence of individual components was confirmed by fluorescence microscopy (Figure 3A). As in single-component spheroids, disruption of spheroid integrity and removal of dead cells was also observed in HCT116wt/MRC5 co-culture spheroids after Prop treatment. On the contrary, treatment with 5-FU activated the formation of new small spheroids detaching from original HCT116wt/MRC5 spheroids. Co-culture spheroids formed from HCT116-FUR/MRC5 and HCT116-PROP/MRC5 cells also showed effects similar to mono-spheroids, with Prop causing pronounced damage to the integrity of spheroids containing 5-FU resistant cells. Growth curves document that Prop, 5-FU, and their combination significantly slow the spheroid growth of all analyzed cell lines (Figure 3B). Treatment with 5-FU caused a stronger growth retardation in HCT116-PROP/MRC5 spheroids compared to single-component spheroids. Similarly, the effect of Prop was stronger in HCT116-FUR/MRC5 spheroids than in mono-spheroids. Immunohistochemical labeling of co-culture spheroids sections showed the CAIX localization profile very similar to that of mono-spheroids. Treatment with 5-FU led to changed localization of CAIX to the surface regions. Similar pattern was visible in spheroids formed from HCT116-FUR (Figure 3C). We analyzed proliferating cells in spheroids through IHC labeling of the nuclear protein Ki67 (Figure 3C). We detected high Ki67 positivity mainly in the surface areas of spheroids with sufficient oxygen diffusion. A marked reduction in Ki67 signal was visible after the action of 5-FU and Prop individually or in combination, whereas a change in the structure of the spheroids and disruption of the integrity of spheroid edges were observed. During migration experiments (Figure 3D,E) Prop inhibited adhesion of spheroids to the substrate for HCT116wt as well as HCT116-FUR co-culture spheroids, Prop also significantly affected migration of cells from spheroids when used in the combination with 5-FU. Spheroids treated by 5-FU alone released apoptotic cells along with migrating cells.

### 2.8. Propranolol Slows the Growth of Xenografts in Nude Mice

**Figure 4:**
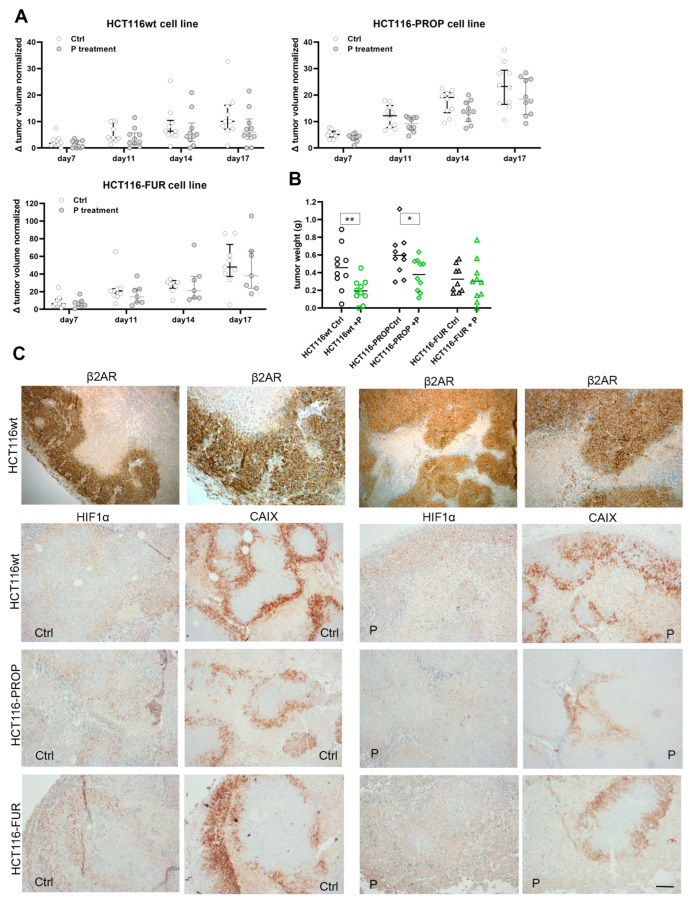
Effect of propranolol on the growth of xenografts formed from parental HCT116 cell line and derived 5-FU resistant and Prop adapted cell lines. (**A**) Growth curves show changes in the volume of separate xenografts untreated/treated by Prop at different timepoints. Xenograft volume was calculated on the basis of caliper measurements. (**B**) Graph comparing weights of xenografts treated/untreated by propranolol. Significance of differences was assessed by *t*-tests. In the graph: * denotes *p* < 0.05, ** *p* < 0.01. (**C**) Representative images of immunohistochemical analysis of β2AR, HIF1α and CAIX of sections of xenografts formed from parental and derived HCT116 cells, control (Ctrl) and treated with Prop (P). Scale bar 100 µm.

To verify the effect of Prop in vivo we monitored the growth of subcutaneous xenografts formed from HCT116, HCT116-FUR and HCT116-PROP cells in nude mice. 10 days after the application of cells, we started to apply Prop i.p. 3 times a week. The control group received PBS 100 μL i.p. The obtained results confirmed the assumptions resulting from in vitro experiments that blocking β-AR has an impact on the progression of tumors. Prop slowed the growth of xenografts from the parental line, but it was also effective in xenografts formed by HCT116-PROP or HCT116-FUR cells (Figure 4A). Xenografts derived from HCT116-FUR cells displayed the highest growth rate. Prop treatment resulted in significantly smaller weights of parental and Prop adapted derived xenografts (Figure 4B). Immunohistochemistry analysis of excised xenografts demonstrates a high level of β2AR receptors with predominant membrane localization (Figure 4C). Propranolol treated xenografts show lower intensity of CAIX staining and higher extent of necrotic regions in accordance with in vitro results. Xenograft formed from HCT116-FUR cells display strong CAIX positivity, on the contrary, HCT116-PROP xenografts exhibit considerably lower intensity of CAIX staining.

## 3. Discussion

Resistance to chemotherapy represents a serious persisting medical problem, ranking among main causes of chemotherapy failure and cancer mortality. In the treatment of CRC, 5-FU is one of the most common and effective chemotherapeutic agents [40]. Resistance of colorectal cancer cells to 5-FU significantly affects the overall survival of CRC patients. The 5-year survival rate for patients with advanced CRC is only 10–15% [41]. Since no adequate alternative modality is available, active search for new drugs and their combinations is needed. There is a possibility to utilize and repurpose already existing therapeutics which were not primarily intended for oncological treatment. Possible use of beta-blockers in oncological treatment has been studied for a long time. Recent studies demonstrate that the overactivation of ARs and signaling dysregulation can promote tumor progression, survival, proliferation, metastatic potential, immune system evasion, tumor angiogenesis and drug resistance [42]. Propranolol, a well-known non-selective beta-blocker, is currently used in the treatment of anxiety, prevention of migraine, has cardioprotective effects and is administered as the first-line medication of infantile haemangiomas (IH) [43]. Thanks to its ability to reduce angiogenesis by blocking VEGFA and HIF1α in IH, its high potential for use in cancer therapy is clearly indicated, since hypoxia and subsequent activation of the expression of HIF-dependent proteins play a vital role in cancer progression. The TME, surrounding malignant tumor cells, is very complex and essential for the tumor growth and progression and for therapeutic resistance [44]. The use of cancer cell monolayers to test the effectiveness of new drugs certainly has its advantages and brings basic information about the effect of potential drugs on cell signaling. Given that the TME is multi-component and that tumor mass has its own spatial architecture, we used mono- and co-culture spheroids to verify our findings. 3D spherical models, which retain in vivo tumor conditions in terms of morphology, functional phenotype, and specialized microenvironments, are an increasingly accepted approach in tumor biology and drug testing [45].

Employing 2D, 3D, and in vivo mouse model, we clearly demonstrate the effect of propranolol on important proteins (CAIX, HIF1α) influencing the tumor microenvironment, activation of apoptosis, growth of spheroids and migration abilities. Furthermore, we show a significant effect of the β-blockade with Prop on the sensitization of 5-FU resistant colorectal cancer cells.

Our results prove that blocking beta-adrenergic signaling with Prop has a profound effect on CAIX protein level. Cells long-term treated with Prop display a significantly lower level of CAIX compared to the parental cells (Figure 1D,E). The level of the regulatory α subunit of a master regulator of hypoxia HIF1, also decreases significantly, which affects adaptation of cells to hypoxic stress (Figure 1D,E). Because an essential hypoxia response pathway is inhibited, the entire cascade of signaling is altered. Our results show that long-term treatment with Prop also reduces the level of other hypoxic targets, e.g., LDHA (Figure 1D,E). This key enzyme involved in pyruvate to lactate conversion, is increased in some types of tumors and correlates with cancer growth, metastasis, tumor recurrence, and poor clinical outcome [46]. Blocking of CAIX and LDHA has a fundamental effect on intratumoral pH and acidification of the extracellular environment, reducing the immunosuppressive nature of the TME and influencing immunotherapy effectiveness [47].

Subsequently, we compared the 5-FU effect on Prop-adapted colorectal cells and sensitive parental cell line. In 2D monolayer, we did not observe any significant changes in HIF1α and CAIX expression after treatment with 5-FU in both HCT116wt and HCT116-PROP. The poor cell response to 5-FU by increasing HIF1 activity in CRC cells was described by Ravizza et al. [48]. Also, Xuan et al. [49] indicated that HIF1α provides a selective advantage in hypoxia by inducing genes associated with angiogenesis and drug transporter proteins and its level can be a predictive factor of the effectiveness of 5-FU-based adjuvant chemotherapy in gastric cancer. However, in the spheroid model with a natural gradient of oxygen and pH, 5-FU treatment resulted in the inhibition of the hypoxic pathway by reducing HIF1α level in HCT116-PROP cells long-term treated with Prop, accompanied by a decrease in CAIX expression (Figure 2A,D). 5-FU thus concomitantly potentiates the effect of Prop.

Prop alone significantly disrupts the coordinated adaptive response to hypoxia. Its effects are enhanced when combined with 5-FU. Combined administration of Prop and 5-FU not only leads to impaired adaptation to hypoxic stress, but at the same time, it disturbs the fragile homeostasis of TME due to inhibited expression of hypoxic targets. This leads to changes in the acidification of the extracellular microenvironment, where CAIX plays an important role [32]. Furthermore, we showed that the concomitant administration of Prop and 5-FU has functional consequences in HCT116-PROP spheroids, such as retardation of spheroid growth and decreased migration ability of spheroid cells (Figure 2B,C,F). These effects can be of fundamental importance for oncotherapy, where inhibition of growth, metastasis, and acidosis is the basic principle of blocking tumor progression [50]. Importantly, changes in TME, reduced extracellular acidification and suppression of hypoxia-driven adaptive mechanisms increase the ability of immune cells to penetrate the tumor, thus improving the effectiveness of immunotherapy [51].

Cancer-associated fibroblasts (CAFs) form an integral part of the TME and support tumor cells by paracrine signaling. Activated CAFs perform regulatory functions in extracellular matrix (ECM) remodeling, cancer cell proliferation, metabolic reprogramming, invasion, stemness, metastasis, immunosuppression and therapy resistance [52,53]. We confirmed our previous findings of impaired adaptation to hypoxia in cells adapted to Prop, manifested by growth slowdown, a very low level of CAIX and a decreased expression of the proliferation marker Ki67 in co-culture spheroid model involving MRC5 fibroblasts (Figure 3A–C). The most pronounced effects were observed in the sensitive parental line treated with the combination of Prop, 5-FU, but also with individual treatments. Previous findings were also confirmed in spheroids exposed to simultaneous long-term β-AR blockade and acute 5-FU administration (Figure 3A–C). Hence, significant effects of Prop and 5-FU are maintained even when the stromal component was included in the TME of spheroid, which indicates a high probability of preserving these effects in vivo.

Treatment of CRC with 5-FU remains among gold standards, but it fails very often due to the development of resistance. To explore possibilities of its overcoming, we prepared HCT116-FUR resistant cells by gradually increasing the concentration of 5-FU (Fi-gure 1A). There are several mechanisms of drug resistance. We detected a high level of TS (Figure 1B), but also increased level of CAIX, a HIF1-target, compared to control HCT116wt cells (Figure 1D,E and Figure 2D). Functional consequence was increased migration of resistant cells from spheroids (Figure 2F). As described by Ravizza et al. [48] and Xuan et al. [49], increased level and activity of HIF1 can be a manifestation of hypoxic tumors resistance to 5-FU.

A characteristic feature of hypoxic tumors is the activation of HIF1 and its targets, aggressive phenotype, poor response to radio- and chemotherapy [54]. We investigated whether the blockade of β-adrenergic signaling by Prop would affect the behavior of resistant HCT116-FUR cells. Our findings in single-component and co-culture spheroids show that Prop disrupts the hypoxic adaptation machinery by inhibiting HIF1α, CAIX, activates apoptosis and thus sensitizes resistant HCT116-FUR cells (Figure 1H and Figure 2A,D). Prop treatment leads to reduced proliferation of resistant cells, retardation of the spheroid growth and slowed down migration (Figure 2B,C,F,G). When fibroblasts were a part of spheroid TME, HCT116-FUR cells treated by Prop were unable to adhere to the solid surface at all (Figure 3D,E). Since increased migration is one of the characteristic features of resistant cells [55,56], its inhibition by a suitable combination of drugs including beta-blockers could fundamentally affect the treatment of resistant CRC tumors. CAIX protein distribution was changed in HCT116-FUR spheroids, we detected CAIX across their entire volume, not only in perinecrotic cells and in areas exceeding the oxygen diffusion capacity (Figure 2D and Figure 3C). The movement of CAIX positive cells to surface areas may also be related to the role of CAIX in migration and metastasis [57,58,59]. By analyzing the influence of Prop on the range of phosphokinases by PPA, we found that Prop activates the phosphorylation of pP70S6K, reduces the level of phosphorylated forms of PRAS40, STAT2, 3, 5, FAK, TOR and of beta-catenin level (Figure 2E). Our results indicate that Prop could affect the entire spectrum of signals involved in tumor cell proliferation, inhibition of apoptosis, promotion of cancer cell stemness, immunosuppression, chemotherapy resistance, tumor invasion [60,61,62,63].

**Figure 5:**
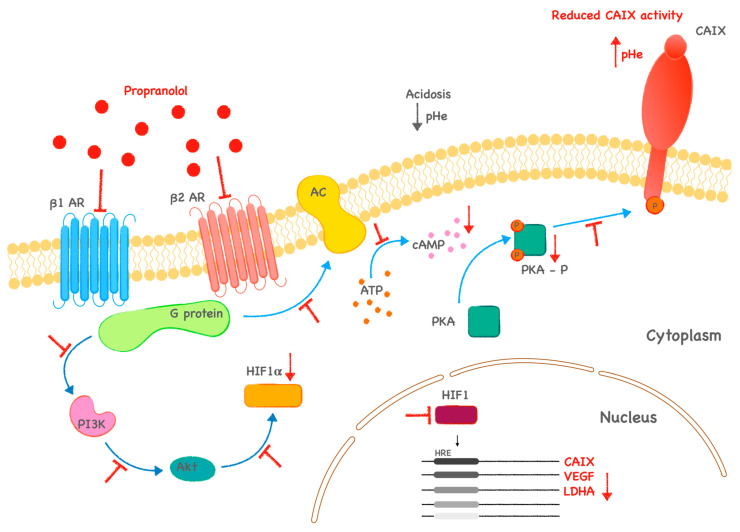
Proposed mechanism of the action of propranolol on tumor cell signaling and processes. Effects of propranolol are marked in red. Cells resistant to 5-FU display high levels of HIF1α and CAIX that play an important role in tumor cell adaptation to microenvironmental condition. Impact of propranolol on cellular adaptation machinery facilitates sensitization of resistant cells to treatment. (**ꓕ**—inhibition, **↓**—decrease).

Our findings of Prop effects were verified in in vivo experiment in mice. We confirmed that Prop slows the growth of xenografts formed from the parental line HCT116 and HCT116-PROP (Figure 4A,B). We also confirmed that Prop sensitizes 5-FU resistant cells and slows down their growth (Figure 4A,B). Our results clearly point to the effect of blocking beta-adrenergic signaling by propranolol not only on CAIX but also on the hypoxic adaptation machinery (Figure 5).

Several studies showed the clinical benefit of Prop in different hyperproliferative diseases and in reduction of biomarkers associated with metastatic potential. Our results revealed that Prop is able to exert its effect not only in chemosensitive colorectal cells, but also in cells already cultured under β-blockade for a long time. At the same time, Prop is able to sensitize 5-FU resistant cells, which may be important in the management of chemo-resistant CRC patients, whose therapy is ineffective, when blockade of AR could be part of treatment strategy.

## 4. Materials and Methods

### 4.1. Cell Culture

Colorectal cancer cells HCT116wt and derived cells (HCT116-FUR, HCT116-PROP), and MRC5 fibroblasts were routinely cultured in DMEM culture medium supplemented with 10% FCS (HyClone Laboratories, Logan, UT, USA) and gentamicin (Sandoz, Basel, Switzerland). Experiments in hypoxia (1% O_2_) were done in a humidified anaerobic workstation (Ruskinn Technologies, Bridgend, UK) with 5% CO_2_, 10% H_2_ and 84% N_2_ at 37 °C. MRC5 was purchased from ATCC, HCT116 cell line was a gift from Dr. Nicholas Denko.

### 4.2. Treatments

Cells were treated with beta-blocker propranolol (Sigma Aldrich, Saint-Louis, MO, USA) at the final concentration 50 µM in DMSO and 5-FU 1 µM in DMSO (Sigma Aldrich, Saint-Louis, MO, USA). Control cells were cultivated in the presence of an appropriate volume of DMSO.

### 4.3. Preparation of the Propranolol-Adapted Cell Line

HCT116wt cells were cultured in DMEM medium supplemented with 50 µM propranolol. After 10 passages cells were considered as adapted to propranolol and denoted as HCT116-PROP.

### 4.4. Preparation of the Cell Line Resistant to 5-FU

5-FU resistant HCT116wt cells were established after sequential treatments with increasing concentration of 5-FU. Initial concentration of 5-FU was 1 µM. Cells cultured in DMEM supplemented with 30 µM 5-FU were denoted as resistant HCT116-FUR.

### 4.5. Formation of Mono- and Co-Culture Spheroids

Monospheroids were prepared according to [35]. Briefly, spheroids were formed in 20 μL hanging drops containing 100 cells/μL in DMEM for 4 days on the lid of Φ100 mm Petri dish, and then transferred to 96-well plates for suspension cells. Co-culture spheroids were formed from the suspension of cancer cells mixed with MRC5 (1:1) in ultra-low attachment surface plates (BIOFLOAT ULA plates, faCellitate, Mannheim, Germany), 2000 cells/100 µL per well. To investigate their composition, both cell lines were pre-stained with CellBrite dyes (Biotium, Fremont, CA, USA) according to manufacturer’s instructions. After formation (4 days), resulting spheroids were cultured in ULA plates with treatments or DMSO (control) for 6 days (or 7 days in case of monospheroids). The spheroids were examined with a Zeiss Axiovert 40CFL microscope, 5× or 10× objective and processed by Axiovision 4.8 software (Carl Zeiss, Jena, Germany).

### 4.6. Antibodies and Chemicals

M75 hybridoma medium [29] (for CAIX detection, WB: 1:3 in blocking buffer, 1 h, RT; IHC: 1:100, 1 h, RT); anti-β-actin (Cell Signaling, Danvers, MA, USA, WB: 1:5000, 1 h, RT); anti-HIF1α (BD Transduction Laboratories, Franklin Lakes, NJ, USA, WB: 1:250, O/N, 4 °C, IHC: 1:50, O/N, 4 °C); anti-cleaved PARP (Cell Signaling, Danvers, MA, USA, 1:1000, O/N, 4 °C); M75 conjugated with Alexa Fluor 555 (IF: 1:250, 1 h, 37 °C); Ki67 (IHC: 1:100, 1 h, RT); propranolol (Sigma Aldrich, Saint-Louis, MO, USA), 5-flurouracil (Sigma Aldrich, Saint-Louis, MO, USA).

### 4.7. Quantitative Real-Time PCR

QPCR protocol was used as previously described in Barathova et al. [35]. Sample Ct values were normalized to β-actin. Relative expression was calculated by ∆∆Ct method. Oligonucleotides used for qPCR were as follows: Thymidylate synthase F: 5′-ACCTGAATCACATCGAGCCA-3′ R: 5′-TTGGATGCGGATTGTACCCT-3′, β-actin F: 5′-CCAACCGCGAGAAGATGACC-3′ R: 5′-GATCTTCATGAGGTAGTCAGT-3′. Amplifications were performed in triplicate and results were calculated from three independent experiments.

### 4.8. Cell Titer Blue

Cell Titer Blue (Promega, Madison, WI, USA) measurement was performed according to the manufacturer’s protocol. HCT116wt and HCT116-FUR cells cultured in hypoxia were treated with 0.01 µM; 0,1 µM; 1 µM; 2 µM; 5 µM; 10 µM; 50 µM; 100 µM 5-FU for 72 h. IC50 value was determined from sigmoidal 4PL curve used for fitting the CTB measurement values of HCT116wt cells treated by 5-FU concentrations. Data interpolation was performed in GraphPad Prism 9.5.1 (Boston, MA, USA).

### 4.9. Evaluation of Cell Proliferation Rate

HCT116wt, HCT116-PROP, and HCT116-FUR cells were seeded into special E-plates (Accela, San Diego, CA, USA) at 5000 cells/well and proliferation was monitored at xCELLigence device (Accela, San Diego, CA, USA) in hypoxia. Doubling times were calculated by RTCA 1.2.1 software. Experiments were performed three times in quadruplicates.

### 4.10. Western Blotting

WB analysis was performed according to Barathova et al. [35], antibodies used are detailed above. Western blot analyses were repeated three times.

### 4.11. Proteome Profiler Array

Proteome profiler array analysis (PPA) was performed using the Human Phosphokinase Array Kit (R&D Systems, Minneapolis, MN, USA, ARY003B) according to the manufacturer’s recommendations. Briefly, HCT116wt spheroids were treated with 50 µM propranolol or appropriate volume of DMSO as control for 5 days. 32 spheroids from each condition were lysed and total proteins were allowed to interact with membranes with spotted antibody arrays overnight at 4 °C. Proteins were quantified by measuring the accumulated pixel density of individual spots and adjusted based on reference spots using ImageJ v1.41g software (NIH, Bethesda, MD, USA).

### 4.12. Immunohistochemical and Immunofluorescence Analysis of Mono- and Co-Culture Spheroids

Spheroids were fixed in 4% PFA and embedded in paraffin. Antigen retrieval was performed at low pH EnVision^®^ Flex Target Retrieval Solution. A Dako EnVision FLEX+ detection system was used for immunohistochemistry staining. Antibodies are detailed above. Stained sections were examined using Leica DM4500B microscope. For CAIX immunofluorescence staining sections were labeled with M75 antibody conjugated with anti-mouse Alexa 555 antibody (Abcam, Cambridge, UK, Unab274040). The nuclei were stained with DAPI (300 nM). After washing the spheroids were analyzed using confocal microscopy LSM510 Meta (Carl Zeiss, Jena, Germany), 20× objective.

### 4.13. Time-Lapse Microscopy

Spheroids treated for 6 days were moved to 12-well adherent plate and left to attach for 3 h. Time-lapse acquisition was performed with a Zeiss Cell Observer System at 10× objective, in the incubation chamber at 37 °C in 21% O_2_, 5% CO_2_ atmosphere. Imaging was managed by Axiovision 4.8 software (Carl Zeiss, Jena, Germany), using Multidimensional Acquisition settings. Transmitted light images were taken every 60 min at min 3 different positions for each sample for 24 h. The migration of cells from a spheroid was measured using ImageJ 1.41g (NIH, Bethesda, MD, USA) as the distance the cells covered for the given period.

### 4.14. In Vivo Experiments

NMRI nude mice (HsdCpb:NMRI-Foxn1nu) were purchased from Anlab (Prague, Czech Republic). Five female animals per group were injected subcutaneously into the flank on both sides with 1 × 10^6^ cells of HCT116wt, HCT116-PROP and HCT116-FUR in 100 μL PBS. All groups were treated 3 times a week starting at day 10 after the implantation. Animals were treated with 100 µL propranolol solution (20 mg/kg, in PBS) in treatment groups and 100 µL PBS for control groups administered i.p. Tumor size was determined by caliper measurements and calculated according to the formula W^2^ × (L/2), where W and L being the tumor width and length, respectively. All animal protocols were approved by the Institutional Ethics Committee of the Biomedical Research Center SAS and the State Veterinary and Food Institute of the Slovak Republic (104/18-221). All animals had access to the food and water ad libitum.

### 4.15. Statistical Analysis

Where applicable results were analyzed by two-tailed unpaired *t*-test, one-way or two-way (growth curves) ANOVA with Dunnett’s multiple comparison test and *p* < 0.05 was considered significant. *p <* 0.05 is denoted as *, *p <* 0.01 as **, *p <* 0.001 as ***. Statistical analysis was performed in GraphPad Prism 9.5.1. (Boston, MA, USA).

## 5. Conclusions

Our results provide clear evidence that β-adrenergic signaling plays an important role in tumors and affects essential signaling pathways modulating the TME and thus the response to anticancer therapy. Prop can thus be repurposed and serve not only as a chemosensitizer, but also as part of a combined therapy aimed at disrupting TME homeostasis.

## Data Availability

The data generated in this study are available upon request from the corresponding author (P.B.).

## Abbreviations

5-FU—5-fluorouracil, AR—adrenergic receptor, CAIX—carbonic anhydrase IX, cPARP—cleaved poly-ADP-ribose polymerase, HIF1—hypoxia-inducible factor 1, IHC—immunohistochemistry, LDHA—lactate dehydrogenase A, Prop or P—propranolol, TME—tumor microenvironment.
